# Selective Control of Eu^3+^ Radiative Emission by Hyperbolic Metamaterials

**DOI:** 10.3390/ma15144923

**Published:** 2022-07-15

**Authors:** Domenico Genchi, Boris Kalinic, Ionut Gabriel Balasa, Tiziana Cesca, Giovanni Mattei

**Affiliations:** NanoStructures Group (NSG), Department of Physics and Astronomy, University of Padua, Via Marzolo 8, 35131 Padua, Italy; domenico.genchi@phd.unipd.it (D.G.); ionutgabriel.balasa@unipd.it (I.G.B.); tiziana.cesca@unipd.it (T.C.); giovanni.mattei@unipd.it (G.M.)

**Keywords:** hyperbolic metamaterials, multilayers, photoluminescence, radiative decay engineering, europium

## Abstract

In recent years the quest for novel materials possessing peculiar abilities of manipulating light at the nanoscale has been significantly boosted due to the strict demands of advanced nanophotonics and quantum technologies. In this framework radiative decay engineering of quantum emitters is of paramount importance for developing efficient single-photon sources or nanolasers. Hyperbolic metamaterials stand out among the best cutting-edge candidates for photoluminescence control owing to their potentially unlimited photonic density of states and their ability to sustain high-k modes that allow us to strongly enhance the radiative decay rate of quantum light emitters. The aim of the present paper is to show how Au/$Al_{2}O_{3}$ hyperbolic multilayers can be used to selectively control the photoluminescence of coupled $Eu^{3+}$ emitters. We point out an enhancement of the $Eu^{3+}$ transitions when they are in the hyperbolic regime of the metamaterials and a significant alteration of the ED and MD branching ratios by changing the emitter–metamaterial distance.

## 1. Introduction

Nowadays, light manipulation at the nanoscale is one of the main requirements of quantum technologies and optical communication [1]. Engineering the photoluminescence (PL) of quantum emitters is one of the critical steps for designing many optoelectronic devices, and represents a fundamental challenge for the development of single-photon sources and nanolasers [2,3]. Consequently, the design and fabrication of novel nanostructured materials able to satisfy this demand has become the greatest purpose of nanophotonics research in the last decade [4]. One of the most efficient ways to control the fluorescence of a light emitter is the tailoring of the photonic density of states (PDOS) available for the emitted photons. A high PDOS gives rise to enhanced spontaneous emission [5]. The PDOS modification can be generally achieved by using resonant nanocavities able to change the optical field density around the emitting system, whose PL is then altered and enhanced [6,7]. However, tricky nanofabrication is required to obtain complex geometries of the cavity structure.

In this context, hyperbolic metamaterials [8] represent a milestone for radiative decay engineering owing to their unique hyperbolic optical dispersion generating from the extreme uniaxial anisotropy of their permittivity tensor, whose diagonal components are opposite in sign ($\epsilon_{xx}\epsilon_{zz}<0$; $\epsilon_{xx}=\epsilon_{yy}\neq \epsilon_{zz}$) beyond the epsilon-near-zero (ENZ) wavelength ($\lambda_{ENZ}$). These properties bring forth a potentially unlimited PDOS and the possibility to sustain the propagation of modes with very large wavevectors (high-k modes) giving hyperbolic media the capability to significantly increase the radiative decay rate of a light emitter in its close proximity (Purcell effect) [9,10,11]. In particular, hyperbolic multilayers (HMs) are very attractive to this scope due to their ability to support surface and bulk plasmon polaritons creating a local enhancement in the PDOS [12,13,14], as well as the simplicity of their fabrication and integration within optical devices, so gaining a great potential as platforms for several advanced applications in quantum nanophotonics, including single-photon sources [15]. Moreover, the properties of hyperbolic metamaterials can be adjusted by changing the composition or the metal filling fraction [16,17], allowing them to provide a huge number of electromagnetic states for light coupling at desired frequencies over a broadband spectral range [9,12], thus achieving PL control for several fluorescent systems, such as dyes [18], perovskites [19], nanoparticles [20] and quantum dots [21].

Among quantum emitters, rare-earth elements are of great interest due to their integration in current technologies and their multilevel luminescence, whose transitions have both an electric dipole (ED) and a magnetic dipole (MD) nature with comparable oscillator strength [22]. Thus, controlling their ED and MD transitions via novel nanostructured materials could help with fully taking advantage of their high luminescence efficiency. Moreover, exploiting the magnetic nature of light emission potentially leads to novel devices and applications [23]. Several studies have been conducted to explain the spontaneous emission behaviour of rare-earth ions near materials surfaces [24,25,26,27,28]. Embedding them in a planar dielectric matrix to be placed near the interface of a planar mirror represents the simplest approach to studying their PL and its dependence on the surrounding PDOS offered by the medium in its vicinity [29]. In particular, organic matrices doped with rare-earth elements are optimal materials for experimentally probing the relative strengths of ED and MD transitions and map the spatial distribution of the optical modes for the spontaneous emission in the system [30]. For instance, europium-doped polymers are among the most appealing for the visible spectrum owing to their easy fabrication, their transparency and, above all, the highly efficient room-temperature photoluminescence of $Eu^{3+}$, whose ED and MD transitions are spectrally close and well-resolved around λ = 615 nm. In recent years, the fluorescence of $Eu^{3+}$ embedded in polymers or glasses has been studied in combination with various systems such as thin films, plasmonic nanostructures or dielectric metasurfaces, pointing out its ED and MD emissions to be adjustable depending on the type or geometry of the underlying material as well as by changing the tilt angle of the sample [31,32,33,34,35].

In the present work, we investigate the room-temperature photoluminescence of $Eu^{3+}$-doped PMMA thin films coupled to two types of Au/$Al_{2}O_{3}$ hyperbolic multilayers with different filling fractions (i.e., different spectral position of their $\lambda_{ENZ}$) by integrated and time-resolved spectroscopy. We point out a significant PL lifetime shortening when the emitter is in close proximity to the metamaterials, and a selective radiative emission enhancement when the $Eu^{3+}$ transitions occur in the hyperbolic regime of the metamaterials. Furthermore, we show the effect of outdistancing the rare-earth element from the metamaterial surface by using a dielectric spacer, demonstrating that the relative intensity of specific MD and ED $Eu^{3+}$ transitions over the full spectrum can be largely altered by changing the distance between the emitter and the HM. In brief, we highlight how HMs can be used to selectively control the PL of a multilevel rare-earth quantum emitter, obtaining an enhancement of its radiative emission when its transitions fall in the hyperbolic regime of the metamaterials, and a significant alteration of the ED and MD branching ratios by changing the emitter–metamaterial distance. This shows the high potential of such systems to be employed as platforms for advanced nanophotonic applications.

## 2. Materials and Methods

### 2.1. Synthesis and Characterization of Hyperbolic Metamaterials

Two types of hyperbolic multilayer metamaterials with different metal filling fractions ($f_{m}$) are produced: HM33 consisting of 15 nm Au layers and 30 nm $Al_{2}O_{3}$ layers ($f_{m}$ = 33%), and HM16 consisting of 16 nm Au layers and 85 nm $Al_{2}O_{3}$ layers ($f_{m}$ = 16%). The layers are arranged in four-period structures with the metal on the top of each period. The constituent materials are chosen due to their high chemical stability and their good interfacial adhesion. The stacks are obtained by alternate magnetron sputtering depositions of gold and alumina thin films in Ar atmosphere ($p=5\times 10^{-3}$ mbar). $SiO_{2}$ overlayers with variable thickness (10 nm, 50 nm, 100 nm, 300 nm) are deposited at the end of the HMs fabrication to be used as spacers. A dielectric material different from the one used in the metamaterial structure is employed for the spacer in order to preserve the HM periodicity and its effective medium properties. A DC source is employed for sputtering the Au target and two RF sources for the $Al_{2}O_{3}$ and $SiO_{2}$ targets. The homogeneity of the depositions is ensured by keeping the sample holder in rotation. The HMs synthesis is performed on monocrystalline Si substrates and on glass slides. Before the depositions, the substrates are cleaned in an acidic piranha solution (30% $H_{2}O_{2}$ + 70% $H_{2}SO_{4}$) at 80 °C for 1 h and rinsed with ultrapure Milli-Q water. The multilayered structure is inspected by cross-section scanning electron microscopy (SEM, Zeiss Sigma HD FE-SEM), and the average surface roughness is measured by atomic force microscopy (AFM, NT-MDT Solver Pro). A spectroscopic ellipsometer (J. A. Wollam WVASE) is employed to measure the thickness of the layers as well as the reflectance and transmittance spectra of the HMs as a function of the incidence angle and polarization. The dielectric functions of the constituent materials are experimentally obtained by characterizing Au thin films (60 nm), $Al_{2}O_{3}$ thin films (30 nm and 85 nm) and $SiO_{2}$ thin films (10 nm, 50 nm, 100 nm, 300 nm) produced by magnetron sputtering in the same deposition conditions employed for the metamaterials synthesis. The Au film thickness is chosen as the sum of the thicknesses of the metal layers in the metamaterials in order to ensure the same absorption given by the metallic component.

### 2.2. Coupling of Emitter-Doped Polymer Films to Hms

Solid polymeric thin films doped with $Eu^{3+}$ emitters are coupled to the produced HMs. The HMs surface is cleaned and hydrophilized by means of an UVO cleaner, then a toluene solution (3 mM) of Europium (III) thenoyltrifluoroacetonate trihydrate ($Eu{\left(\mathrm{TTA}\right)}_{3}\cdot 3H_{2}O$) and polymethyl methacrylate (PMMA, 1% *w*/*w*) is spin coated on their top. The spin coating is performed at 3000 rpm for 1 min at room temperature, conditions determined as optimal to obtain homogeneous films with controlled reproducible thickness (30 nm, within 5% error). The emitter organic complex and the polymer are commercially available from *Acros Organics* and *Sigma-Adrich* respectively. Thermal curing at T = 125 °C for about 30 min is performed right after the spin coating deposition in order to evaporate the residual solvent and make the doped layer solidify and harden. Its thickness is verified by AFM and ellipsometry. Reference samples of doped films with the same thickness are produced in identical conditions on Au thin films (60 nm thick), Si and $SiO_{2}$ substrates. Doped films with three different emitter concentrations are made on Si and $SiO_{2}$ substrates starting from solutions with concentrations 0.15 mM, 0.3 mM and 3 mM. These respectively correspond to a percent molar fraction of *x* = 0.6%, *x* = 1.2% and *x* = 11%. The absorbance spectrum of the doped polymer on the reference $SiO_{2}$ substrates is measured by a *Jasco V670* UV-VIS spectrophotometer.

### 2.3. Integrated and Time-Resolved Photoluminescence Measurements

Integrated and time-resolved PL measurements are conducted at room temperature in order to study the emission properties of the $Eu^{3+}$ emitters coupled to our HMs. The third harmonic (λ = 355 nm) of an ns-pulsed Q-switched Nd:YAG laser (Brilliant by Quantel Laser) is employed as the excitation light source. The pulses have a duration of 5 ns and a repetition rate of 10 Hz. The output beam intensity is attenuated by optical density filters to prevent photobleaching. The laser beam impinges on the sample at an angle of 30°. The sample is positioned orthogonally with respect to the direction of the photodetection system. The emitted light is collected by a converging lens with numerical aperture NA = 0.26 within an angle of 28°. The collected light is collimated and then focused by another converging lens onto the entrance slit of single-grating monochromator, preceded by a longpass filter (λ>550 nm) to avoid scattered laser light to enter the detector. A near-infrared photomultiplier tube (HAMAMATSU R5509-72) cooled by liquid nitrogen is employed as a photodetector, connected to a Tektronix TDS7104 Digital Phosphore Oscilloscope and a lock-in amplifier.

### 2.4. Simulation Methods

The optical properties (reflectance and transmittance) of the fabricated samples and the electromagnetic field distribution inside them are calculated by the scattering matrix method via EMUstack [36]. COMSOL Multiphysics is employed to perform finite element method (FEM) simulations of the far-field irradiance angular distribution for an emitting dipole coupled to the hyperbolic metamaterials. In both cases the HMs are modeled as their actual layered structure.

## 3. Results and Discussion

### 3.1. Structural and Optical Properties of the Hyperbolic Metamaterials

**Figure 1:**
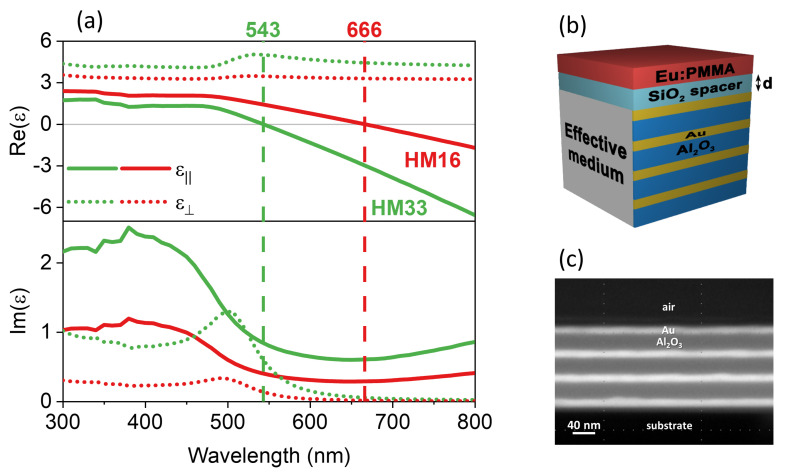
(**a**) In-plane ($\epsilon_{\|}$, solid) and out-of-plane ($\epsilon_{⊥}$, dotted) components of the effective permittivity (real part in the upper panel, imaginary part in the lower panel) of the produced hyperbolic metamaterials. (**b**) Sketch of the hyperbolic multilayer with $SiO_{2}$ spacer (of thickness *d*) and $Eu^{3+}$-doped PMMA film on top. (**c**) SEM image of HM33 with no spacer.

Two types of Au/$Al_{2}O_{3}$ HMs are produced (see Section 2). The structure is chosen in order to obtain their ENZ wavelength in the visible range. The in-plane and out-of-plane components of the effective permittivity are calculated according to the effective medium theory [37]:

(1) $$\epsilon_{\|}=\epsilon_{xx}=\epsilon_{yy}=f_{m}\epsilon_{m}+\left(1-f_{m}\right)\epsilon_{d}$$

(2) $$\frac{1}{\epsilon_{⊥}}=\frac{1}{\epsilon_{zz}}=\frac{f_{m}}{\epsilon_{m}}+\frac{\left(1-f_{m}\right)}{\epsilon_{d}},$$

by using the permittivities of the metal $\epsilon_{m}$ and the dielectric $\epsilon_{d}$. These are experimentally obtained by ellipsometric measurements performed on reference samples as described in Section 2, and they are reported in Figure S1 of the Supplementary Materials. The measured values are in good agreement with tabulated ones [38,39,40]. The metal filling fraction is calculated as $f_{m}=t_{m}/\left(t_{m}+t_{d}\right)$, where $t_{m}$ and $t_{d}$ are the thicknesses of the metal and the dielectric layers respectively. The filling fraction is tuned by changing the thickness of the dielectric layers, and it is selected to be inside an optimal range of values ($f_{m}=0.05-0.35$) to obtain a high Purcell factor for the $Eu^{3+}$ radiative transitions [35]. The real and imaginary parts of the permittivity components for the two HMs are displayed in Figure 1a. $Re\{\epsilon_{\|}\}$ exhibits the zero-crossing at $\lambda_{ENZ}$ = 543 nm for HM33 and $\lambda_{ENZ}$ = 666 nm for HM16. $Re\{\epsilon_{⊥}\}$ has a positive sign in the whole spectrum. When $\epsilon_{\|}\epsilon_{⊥}<0$ (i.e., for $\lambda >\lambda_{ENZ}$), the metamaterial exhibits its hyperbolic dispersion, whereas elliptical dispersion is obtained before $\lambda_{ENZ}$. $Im\{\epsilon_{\|}\}$ and $Im\{\epsilon_{⊥}\}$ have the same spectral shape for both the metamaterials and are modulated in absolute value, being inversely proportional to the amount of alumina in the HM. A sketch of the hyperbolic multilayers is depicted in Figure 1b and the SEM image in Figure 1c shows the cross-section of HM33 as an example. The sketch also includes a $SiO_{2}$ spacer (of thickness *d*) and the $Eu^{3+}$-doped PMMA film (Eu:PMMA) deposited on the top of the metamaterials for the photoluminescence study described in Section 3.2. The surface roughness of the samples measured by AFM is equal to 1.3 ± 0.1 nm. The reflectance and transmittance spectra of the fabricated HMs are calculated and experimentally measured as a function of angle and polarization: in Figure S2 of Supplementary Materials we report the optical properties at 30° with *p*-polarization (namely the angle and polarization of the incident laser beam used for the PL measurements) and we compare them with those of an Au thin film reference sample. The EMUstack calculations are performed considering the actual layered structure of the HMs. The good quality of the fabricated stacks is evidenced by the low surface roughness, the sharp contrast between Au and $Al_{2}O_{3}$ layers in the SEM image, and the good agreement between the experimental and calculated optical spectra.

### 3.2. Photoluminescence of $Eu^{3+}$ Emitters Coupled to the Hyperbolic Metamaterials

**Figure 2:**
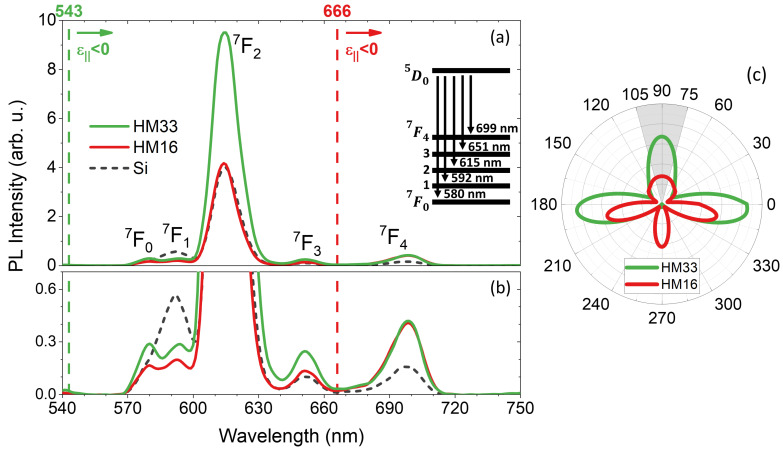
(**a**) Photoluminescence spectra of $Eu^{3+}$ embedded in a PMMA thin film deposited on the hyperbolic multilayers and the reference Si substrate, all having a 10 nm spacer on top. The vertical dashed lines indicate the ENZ wavelength of each metamaterial and the arrows indicate the spectral region of their hyperbolic regime. Inset: energy-level diagram of the $\mathrm{Eu}{\left(\mathrm{TTA}\right)}_{3}$ radiative decay channels between the excited state ${}^{5}D_{0}$ and the ${}^{7}F_{J}$ levels. (**b**) Magnification of the less intense radiative transitions. (**c**) Angular distribution of far-field irradiance (in W/m${}^{2}$) at λ = 615 nm simulated by COMSOL for an emitting electric dipole in close proximity of HM33 (green) and HM16 (red) with a silica spacer (10 nm) on top. The gray area indicates the collection angle of our experiment.

We investigate the photoluminescence properties (PL intensity and lifetime) of $Eu^{3+}$-doped polymeric films coupled to the hyperbolic metamaterials (see sketch in Figure 1b). The $\mathrm{Eu}{\left(\mathrm{TTA}\right)}_{3}$ organic complex is chosen because it exhibits a highly efficient room temperature fluorescence in the visible spectrum with well resolved ED and MD dipole transitions, and it can be easily embedded within a polymer matrix, thus representing a good candidate for solid-state and flexible devices. Before studying the behaviour of $Eu^{3+}$ coupled to the HMs, a preliminary characterization of the reference samples (Eu:PMMA deposited on Si and $SiO_{2}$ substrates) is performed in order to select the optimal concentration of $Eu^{3+}$ emitters within the polymer matrix for obtaining a revealable PL signal avoiding concentration quenching. This is confirmed by the linear trend of the PL signal as a function of the dopant concentration displayed in Figure S3a,b of the Supplementary Materials. The same figure (panel c) shows the absorbance spectra of the $\mathrm{Eu}{\left(\mathrm{TTA}\right)}_{3}$-doped PMMA films as a function of the molar fraction. The distinctive absorbance band of $\mathrm{Eu}{\left(\mathrm{TTA}\right)}_{3}$ around 346 nm is observed, and the peak value linearly increases with the emitter concentration as expected. The PL study in combination with the HMs is conducted with a doping concentration of *x* = 11% in order to obtain a well-revealable PL intensity with a high signal-to-noise ratio and thus to clearly distinguish all the transitions. In Figure 2a,b we depict the measured PL spectra of Eu:PMMA coupled to the sample HM16 (solid red) and HM33 (solid green) as well as to the reference Si substrate (dashed gray). All the samples have a 10 nm silica spacer on their top. Five emission peaks are revealed in the spectra, which correspond to the $Eu^{3+}$ radiative transitions according to its multilevel diagram (see inset in Figure 2a). By using the Russel–Saunders notation (${}^{2S+1}L_{J}$), the peak at 592 nm corresponds to the ${}^{5}D_{0}\to {}^{7}F_{1}$ transition and it has a magnetic dipole (MD) nature; instead the peaks at 580 nm, 615 nm, 651 nm and 699 nm have an electric dipole (ED) nature and they correspond to the transitions from the ${}^{5}D_{0}$ excited state to the states ${}^{7}F_{0}$, ${}^{7}F_{2}$, ${}^{7}F_{3}$ and ${}^{7}F_{4}$ respectively [22,32].

By considering the ENZ properties of the metamaterials, the $Eu^{3+}$ transitions can fall in two different regimes (hyperbolic when $\varepsilon_{\|}<0$ or elliptical when $\varepsilon_{\|}>0$) depending on the underlying HM and the spectral position of $\lambda_{ENZ}$ (indicated by the vertical dashed lines in Figure 2a,b). The metal filling fraction of the HMs is specifically chosen to accommodate all the $Eu^{3+}$ transitions in the hyperbolic regime in one case (HM33) and in the elliptical regime (except the ${}^{5}D_{0}\to {}^{7}F_{4}$ transition) in the other one (HM16). The PL measurements performed on the two considered emitter-HM systems in identical conditions highlight a different effect of each multilayer on the $Eu^{3+}$ fluorescence, demonstrating that the PL of a light emitter can be modified by tailoring the permittivity of the underlying HM, that is by moving the spectral position of the ENZ wavelength. In particular, the far-field outcoupling efficiency for specific transitions of a multilevel emitting system can be selectively boosted when they fall in the hyperbolic regime of the HMs. As a matter of fact, when Eu:PMMA is deposited on HM33 the main transition at 615 nm (${}^{5}D_{0}\to {}^{7}F_{2}$) is in the hyperbolic regime and its emission peak is 2.5 times higher than when the emitter is coupled to HM16, with which the transition is in the elliptic regime. This is quantitatively consistent with the FEM simulations in Figure 2c, performed by COMSOL considering the actual layered structure of the HMs, and showing the angular distribution of the far-field irradiance for an electric dipole with isotropic orientation with respect to the interface of HM33 and HM16: the graph points out a different emission efficiency inside the collection angle of our experiment (gray area) with the two HMs (higher with HM33 by a factor 2.5), and it evidences the angular dependence of the photoemission due to the presence of the interface [25]. A similar intensity augmentation occurs for the transitions at 580 nm, 592 nm and 650 nm, which are twice stronger with HM33 rather than with HM16. On the contrary, with both the considered metamaterials the same PL intensity is measured for the ${}^{5}D_{0}\to {}^{7}F_{4}$ transition at λ = 699 nm since it falls in their hyperbolic regime in both cases. Moreover, by comparing the spectra of Eu:PMMA deposited on the HMs and on the reference silicon, it is also worth noting that the transitions ${}^{5}D_{0}\to {}^{7}F_{0}$ at 580 nm and ${}^{5}D_{0}\to {}^{7}F_{1}$ at 592 nm are well separated when Eu:PMMA is coupled to the HMs. In particular, HM33 is able to intensify the former transition which is generally very low or completely shadowed by the latter. The spectra also suggest that the PL enhancement depends on the nature of the transitions (ED or MD), and especially that the ED transitions of $Eu^{3+}$ are favoured when they occur in the hyperbolic regime of the closely coupled HMs. For example, with HM16 the emission intensity for all the ED transitions at $\lambda <\lambda_{ENZ}$ is comparable to that obtained with Si, whereas the intensity of the ED transition at 699 nm (i.e., at $\lambda >\lambda_{ENZ}$) is increased by a factor of 2.5.

**Figure 3:**
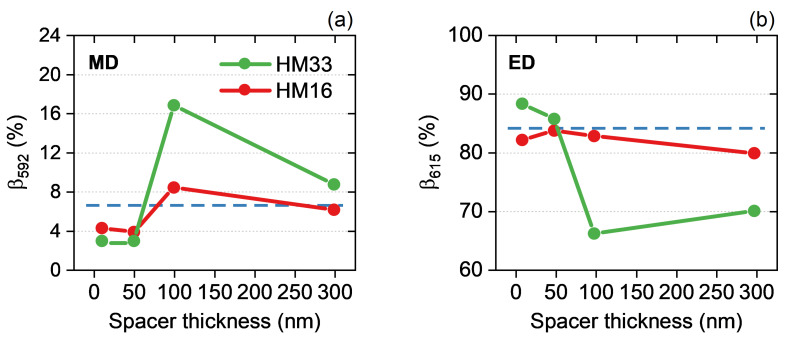
Experimental branching ratio of (**a**) the ${}^{5}D_{0}\to {}^{7}F_{1}$ transition at λ = 592 nm (MD) and (**b**) the ${}^{5}D_{0}\to {}^{7}F_{2}$ transition at λ = 615 nm (ED) calculated as a function of the spacer thickness. The horizontal blue dashed lines represent the BR calculated for each transition with Eu:PMMA deposited on a $SiO_{2}$ slide.

Another parameter indicating the selectivity of the PL emission is the fluorescence branching ratio (BR, β). This is defined as the ratio between the integrated intensity at the specific wavelength of a J-th transition and the integrated intensity taken over the full spectral range:

(3) $$\beta_{J}=\frac{I_{J}}{\sum_{J}I_{J}},$$

which can be assumed to be proportional to the radiative decay rate within the far-field light collection angle defined by the NA of the collecting lens ($I_{J}\propto \Gamma_{r,J}^{\left(NA\right)}$) [31]. In Figure 3 we report the percent branching ratio calculated for the transitions ${}^{5}D_{0}\to {}^{7}F_{1}$ at λ = 592 nm (MD) and ${}^{5}D_{0}\to {}^{7}F_{2}$ at λ = 615 nm (ED) as a function of the spacer thickness. Indeed, it is known that the radiative decay rate of a light emitter can be modulated by changing its distance from an interface [5,24,29]. Therefore, we perform PL measurements on Eu:PMMA films deposited on HM33 and HM16 using a $SiO_{2}$ layer with a different thickness *d* (10 nm, 50 nm, 100 nm, 300 nm) between the doped polymer and the metamaterial, and we determine $\beta_{592}$ and $\beta_{615}$ at each distance. The use of a spacer above the HMs is also expected to bring an advantage in terms of PL enhancement due to a lower non-radiative energy transfer that could be provoked by the direct contact of Eu:PMMA with the top gold layer in the metamaterials [24,27]. In Figure S4 of Supplementary Materials, we depict the PL spectra of Eu:PMMA deposited on HM33 and HM16 as a function of the spacer thickness compared to the reference PL spectrum of Eu:PMMA on the silica slide. In all the analyzed cases, the BR is importantly altered by the combination of Eu:PMMA with the HMs and oscillations with changing emitter–interface distance are observed. Within the self-interference theory these are explained as variations of the radiative decay rate within the collection angle $\Gamma_{r}^{\left(NA\right)}$, which are derived in terms of fields reflected by the interface: when the reflected field is in phase with the emitted field at the dipole location $\Gamma_{r}^{\left(NA\right)}$ is enhanced; conversely, when it is out of phase $\Gamma_{r}^{\left(NA\right)}$ is inhibited [5,24,29]. The MD transition at 592 nm (panel a) is strongly enhanced when the emitter is 100 nm far from the HMs. In particular, with HM33 (HM16) a four-fold (two-fold) increase of $\beta_{592}^{MD}$ is obtained as compared to when a spacer with smaller thickness is employed. At the same distance an enhancement by a factor of 2.5 is obtained with HM33 against the silica reference (blue dashed line). Far from the interface the BR tends to the value of Eu:PMMA on the silica substrate, that is the limit case of the absent HM interface. The opposite trend in the BR is observed when the ED transition at 615 nm is considered (panel b). This is a consequence of the π-phase difference between the reflected electric and magnetic fields [24]. With HM33, $\beta_{615}^{ED}$ rapidly drops by 30% in the first 100 nm. Conversely, with HM16 the BR keeps quite stable around 82%, a value comparable with that obtained on silica (84%). The described study shows that the alteration of both the MD and ED branching ratios is stronger with HM33 rather than with HM16 because in the former case the transitions fall in the hyperbolic regime of the metamaterials whereas in the latter one they are in the elliptic regime. This further corroborates the capability of hyperbolic metamaterials to selectively enhance specific ED or MD transitions of a multilevel emitting system by tuning the ENZ wavelength as well as by changing the distance of the light emitter from the stack interface.

**Figure 4:**
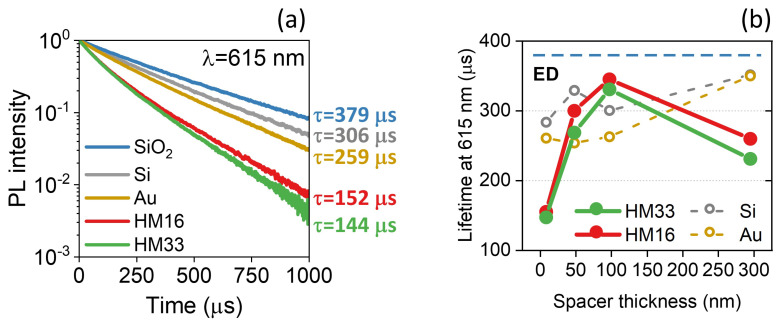
(**a**) Photoluminescence decay curves and lifetimes measured for the ${}^{5}D_{0}\to {}^{7}F_{2}$ transition (λ = 615 nm) of $Eu^{3+}$ coupled to the fabricated samples (HM33, HM16, Au thin film, Si substrate, $SiO_{2}$ slide). HM33, HM16, Au and Si have a 10 nm $SiO_{2}$ spacer on top. (**b**) Photoluminescence lifetime of $Eu^{3+}$ coupled to the hyperbolic multilayers as a function of the $SiO_{2}$ spacer thickness. The results are reported for the ${}^{5}D_{0}\to {}^{7}F_{2}$ transition (λ = 615 nm, and they are compared to those obtained for the reference samples (Si and Au). The horizontal blue dashed line indicates the lifetime measured for a reference sample made up of Eu:PMMA on a $SiO_{2}$ slide.

Finally, we perform time-resolved PL measurements on Eu:PMMA coupled to the HMs and the reference substrates (Au, Si and $SiO_{2}$) at the wavelengths of the main transitions: λ = 592 nm (${}^{5}D_{0}\to {}^{7}F_{1}$, MD), λ = 615 nm (${}^{5}D_{0}\to {}^{7}F_{2}$, ED), and λ = 699 nm (${}^{5}D_{0}\to {}^{7}F_{4}$, ED). In Figure 4a we report the PL decay curve and the value of the PL lifetime (τ) measured at 615 nm for each sample. Since the $Eu^{3+}$ emission shows a non-single exponential decay, probably ascribed to local inhomogeneities of the polymer matrix around the $Eu^{3+}$ ions and to a non-uniform distance of the emitters from the interface, τ is an effective lifetime estimated as [41,42]:

(4) $$\tau =\frac{\tau_{d}}{k_{s}}\Gamma \left(\frac{1}{k_{s}}\right),$$

where Γ is the Euler’s gamma function and $\tau_{d}$ is the PL lifetime extracted by fitting the experimental decay signal with a stretched exponential function (Kohlrausch–Williams–Watts function):

(5) $$I\left(t\right)=I_{0}\exp{\left(-\frac{t}{\tau_{d}}\right)}^{k_{s}},$$

with I(t) being the time-dependent intensity, $I_{0}$ the intensity at time *t* = 0 and $k_{s}$ the stretching factor ranging from 0 to 1. The results reveal an important shortening of the PL lifetime when the $Eu^{3+}$ emitters are coupled to the HMs: with both HM33 (green curve) and HM16 (red curve), τ is roughly 2.5 times shorter than that measured with Eu:PMMA deposited on a dielectric substrate (silica, blue curve). A strong decay rate enhancement is also observed with respect to the cases of Eu:PMMA on a semiconductor substrate (silicon, gray curve) or a gold thin film (60 nm thick, gold curve). The same effect occurs at each considered wavelength (see Figure S5a,b of Supplementary Materials). The registered lifetime shortening evidences the high efficiency of the coupling between $Eu^{3+}$ and the HMs, and can be explained in terms of strong PDOS enhancement inside and in very close vicinity to the hyperbolic metamaterials [9,12]. It is worth stressing that the PL decay rate of Eu:PMMA coupled to the HMs is two times stronger than in the presence of an optically thick Au film (thickness 60 nm). The data suggest that the first 15-nm-thick Au layer in the metamaterials plays a crucial role in the PDOS modification. Figure S6 of the Supplementary Materials displays the radiative decay rate enhancement computed at λ = 615 nm with the classic dipole oscillator (CDO) model [6] as a function of the thickness of an Au film. The modeled system (see inset of Figure S6) takes into account an ED emitter near a single period of the metal-dielectric multilayer (i.e., 1 Au layer + 1 $Al_{2}O_{3}$ layer). A higher radiative decay rate enhancement is calculated for ED emitters near a thin Au layer (thickness 10–15 nm) compared to those obtained for an optically thick Au film. Furthermore, in Figure 4b we show the lifetime measured at λ = 615 nm as a function of the silica spacer thickness *d*. For each sample, τ reveals an oscillating trend as a function of the emitter–interface distance, as expected from the self-interference theory [5,24]: the radiative decay rate is significantly modified in close proximity to the interface and it gradually tends to the limiting case of emission without interface by increasing the distance. In particular, with both the HMs the lifetime varies from a minimum value of about 150 μs with the 10-nm-thick spacer to a value almost three times higher for *d* = 100 nm and then slightly decreases at the largest distance (*d* = 300 nm). A very different trend is obtained with Au and Si: τ has a less pronounced modulation as a function of the emitter–interface distance, i.e., it varies in the 250–350 μs range. At *d* = 300 nm the emitter–interface coupling is less efficient and the lifetime has the same value with both substrates, whereas larger discrepancies are observed close to them due to a stronger self-interference effect. In all the considered configurations τ remains lower than the value measured on the reference sample (Eu:PMMA on silica slide, horizontal blue dashed line). No significant differences in the trends of τ as a function of *d* are observed at the other considered wavelengths (see Figure S5c,d), i.e., the lifetime shortening does not depend on the nature of the transition. The obtained results highlight that an important lifetime variation can be obtained by outdistancing the light emitter from the HM’s surface, and the alteration of τ is much stronger in presence of the HMs rather than a single Au film or a Si substrate, confirming the the great ability of such metamaterials to control the radiative decay rate of a nearby emitter.

## 4. Conclusions

We show how Au/$Al_{2}O_{3}$ hyperbolic multilayers can be employed to selectively control the photoluminescence and enhance the radiative decay rate of $Eu^{3+}$ emitters embedded in a polymeric overlayer by tuning the metamaterial’s ENZ wavelength or changing the emitter–metamaterial distance. We evidence a selective increase of the photoluminescence intensity when the emitter transitions fall in the hyperbolic regime of the metamaterials and a remarkable shortening of the excited state lifetime when $Eu^{3+}$ is in their close proximity. We point out an important alteration of the transition’s branching ratio by changing the distance between the emitter and the multilayer depending on the nature of the transition. The obtained results highlight the great potential of hyperbolic multilayers for PDOS engineering and, combined with their easy fabrication and integrability with existing technologies, their applicability as platforms for advanced applications in nanophotonics such as single-photon sources and nanolasing devices.

## Supplementary Materials

The following are available online at https://www.mdpi.com/article/10.3390/ma15144923/s1, Figure S1: Permittivity of the multilayers constituent materials; Figure S2: Transmittance and reflectance of the hyperbolic metamaterials; Figure S3: PL spectra as a function of the emitter concentration; Figure S4: PL spectra of Eu:PMMA on HM33 and HM16 as a function of the spacer thickness; Figure S5: PL lifetimes at 592 nm and 699 nm; Figure S6: Radiative decay rate enhancement as a function of the Au layer thickness.
