# Supplementary material for: Selective Control of Eu3+ Radiative Emission by Hyperbolic Metamaterials
Source: Materials (Basel). 2022 Jul 15;15(14):4923. doi: 10.3390/ma15144923 (PMC9322813; doi:10.3390/ma15144923)
Supplement: Supplementary file 1 [file materials-15-04923-s001.zip › materials-1807500-supplementary.pdf]

Supplementary Materials: Selective Control of Eu^{3+} Radiative Emission by Hyperbolic Metamaterials

Domenico Genchi, Boris Kalinic *, Ionut Gabriel Balasa, Tiziana Cesca and Giovanni Mattei

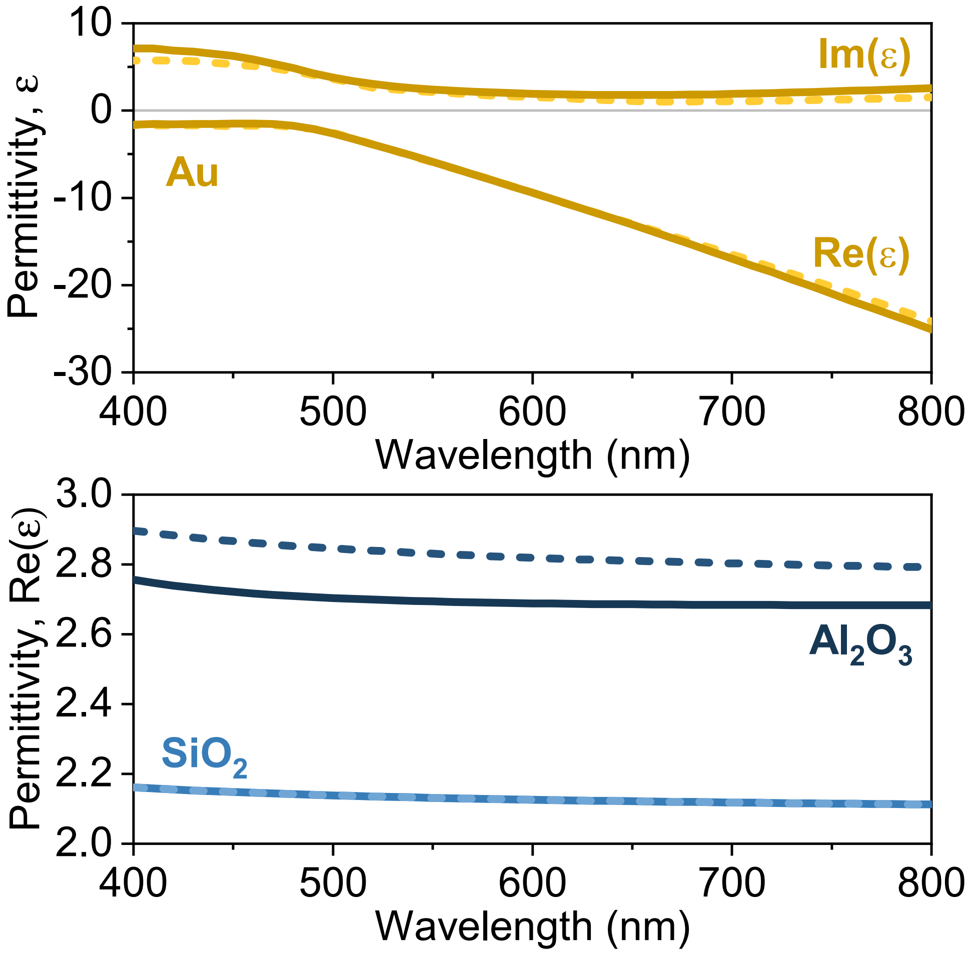

Figure S1. Permittivity of the multilayers' constituent materials. The experimental curves (solid lines) obtained by spectroscopic ellipsometry are compared with tabulated data (dashed lines; Au: Johnson and Christy, Phys. Rev. B, 6, 4370 (1972); Al_2O_3: Boidin et al., Ceram. Int., 42, 1177 (2016); SiO_2: Palik, Handbook of optical constants of solids; Vol. 3, Academic Press, Elsevier, 1998).

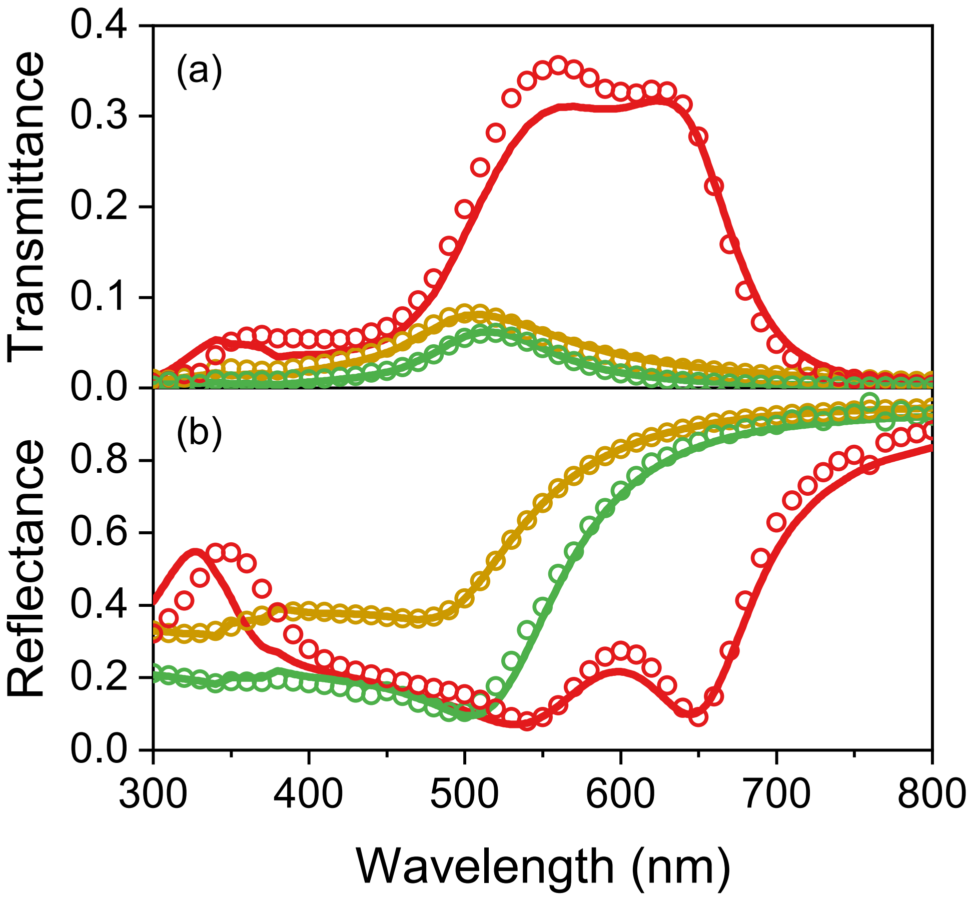

Figure S2. (**a**) Transmittance and (**b**) reflectance spectra of HM33 (green), HM16 (red) and Au thin film (60 nm, gold) at 30° with *p*-polarization. The solid lines are calculated by EMUstack, while the open symbols are the experimental data obtained by ellipsometry.

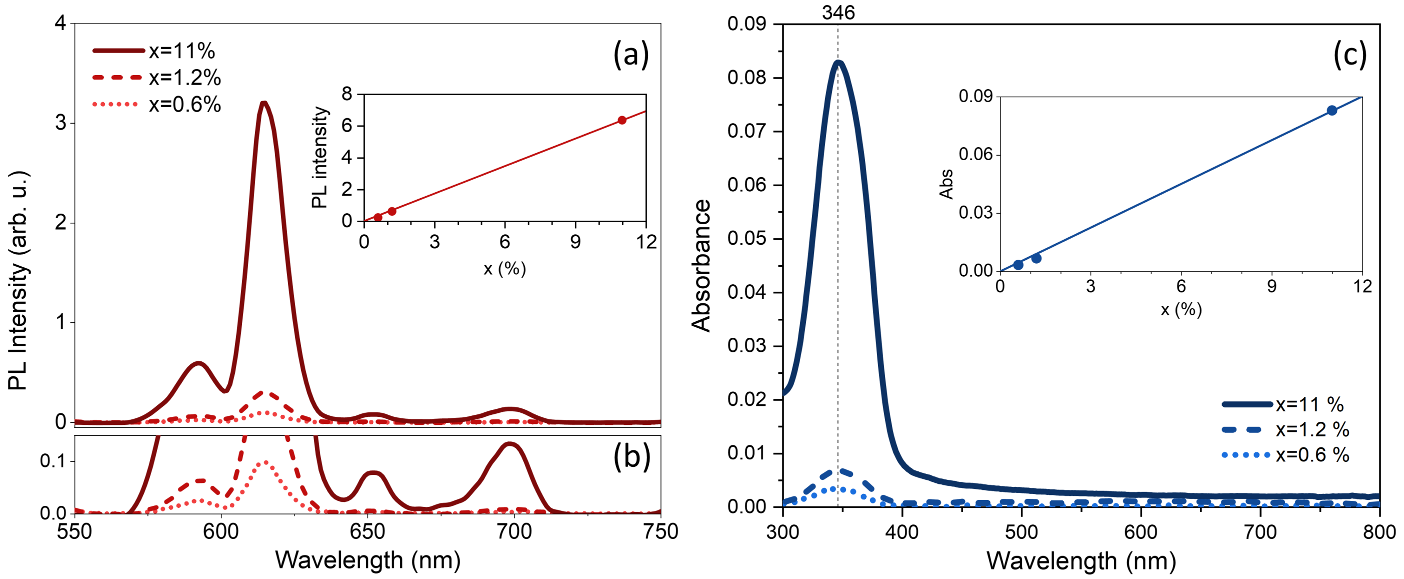

Figure S3. (**a**) PL spectra of Eu:PMMA on Si as a function of the emitter concentration and (**b**) magnification of the transitions with lower intensity. The inset plot shows the linear trend of the PL intensity (calculated as the integral of the spectra) as a function of the molar fraction. (**c**) Optical absorbance spectra of Eu(TTA)$_3$ in PMMA (1% w/w) with different molar fractions in form of a solid thin film (30 nm thick) spin-coated on a SiO_2 substrate. The spectra are acquired by a *Jasco V670* spectrophotometer. The absorbance of an undoped 30 nm thick PMMA thin film produced in identical conditions is used as baseline. The inset graph shows the linear dependence of absorption (peak value at 346 nm) on concentration; the error bars (5%) are not visible since smaller than the point size.

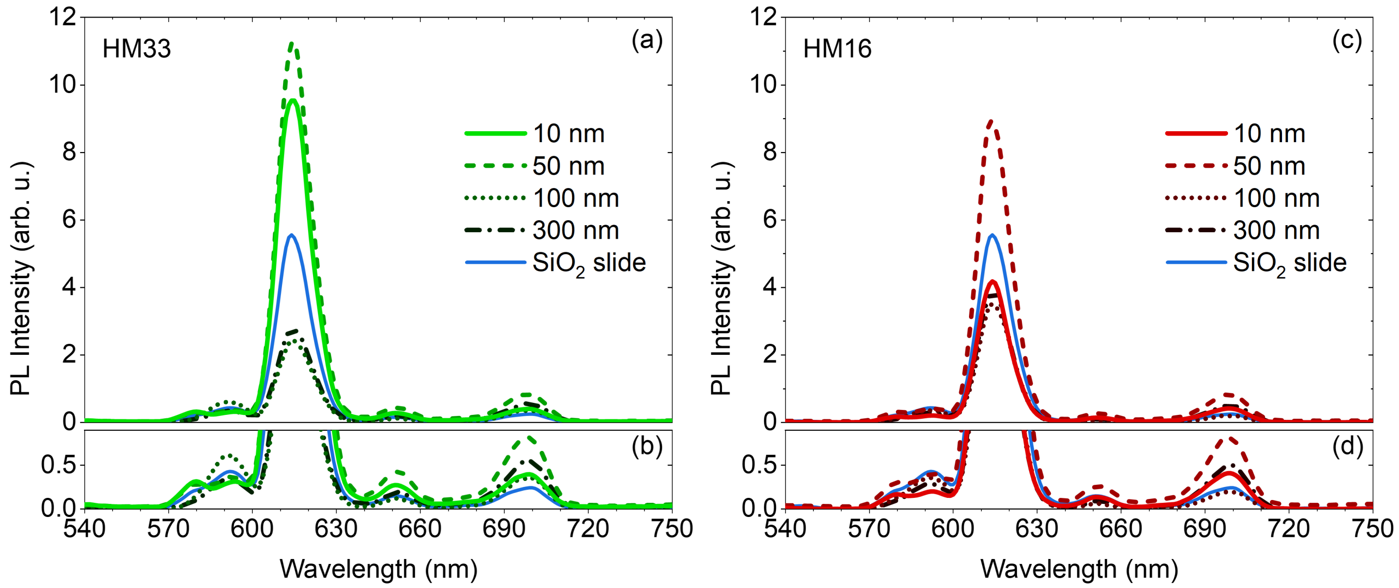

Figure S4. Photoluminescence spectra of (**a**,**b**) HM33 and (**c**,**d**) HM16 as a function of the SiO_2 spacer thickness deposited on their top. The blue solid curves indicate the spectra of Eu:PMMA deposited on a silica slide as a reference.

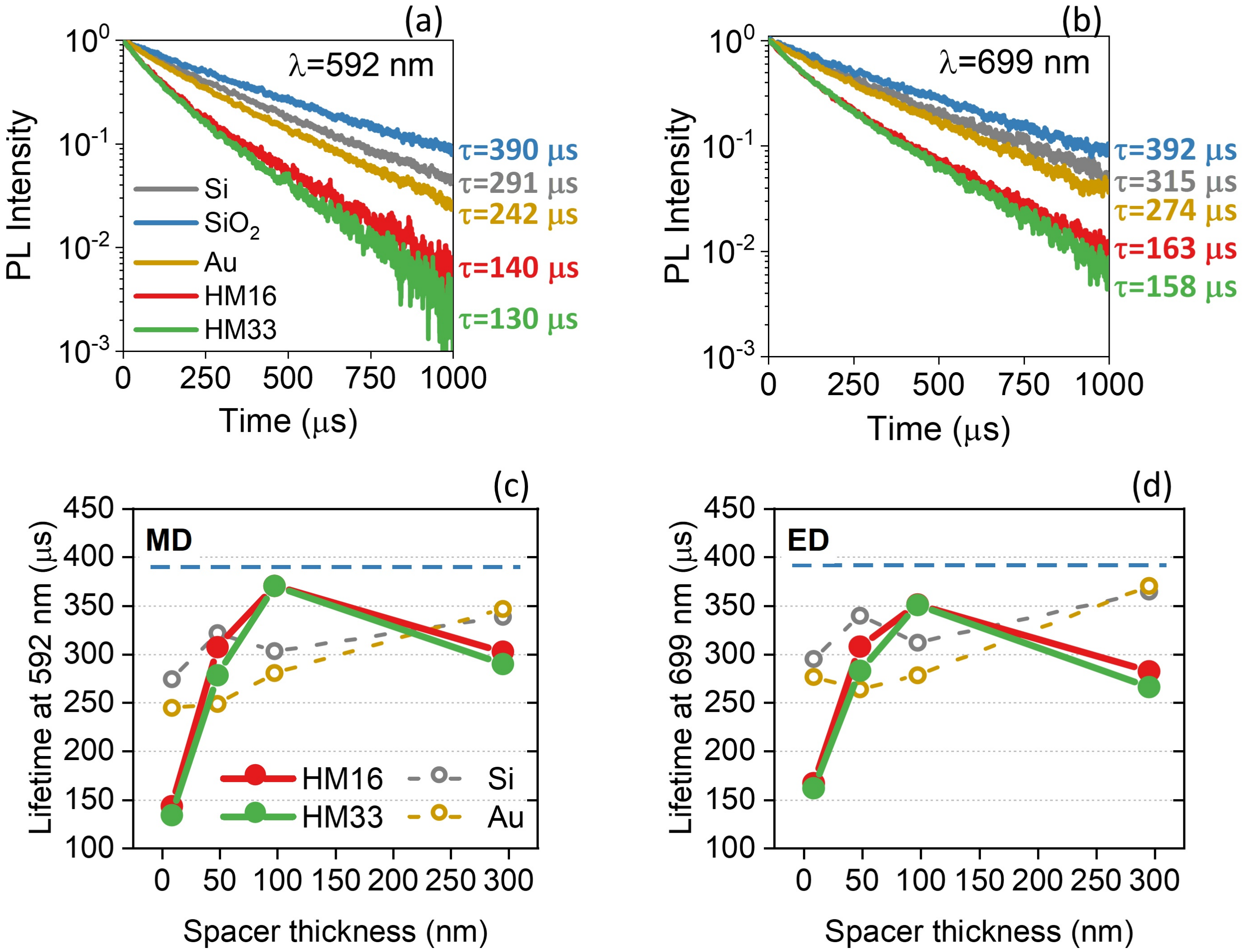

Figure S5. (**a**,**b**) Photoluminescence decay curves and lifetimes measured for the (**a**) $^5D_0 \rightarrow {}^7F_1$ (λ = 592 nm) and (**b**) $^5D_0 \rightarrow {}^7F_4$ (λ = 699 nm) transitions of Eu^{3+} coupled to the fabricated hyperbolic multilayers and reference samples (Au thin film, Si substrate, SiO_2 slide). (**c**,**d**) PL lifetime of Eu^{3+} coupled to the HMs as a function of the SiO_2 spacer thickness. The results are reported for (**c**) the $^5D_0 \rightarrow {}^7F_1$ (λ = 592 nm) and (**d**) the $^5D_0 \rightarrow {}^7F_4$ transitions (λ = 699 nm), compared to those obtained for the reference samples (Si and Au substrates). The horizontal blue dashed line indicates the PL lifetime measured for a reference sample made up of Eu:PMMA on a SiO_2 slide.

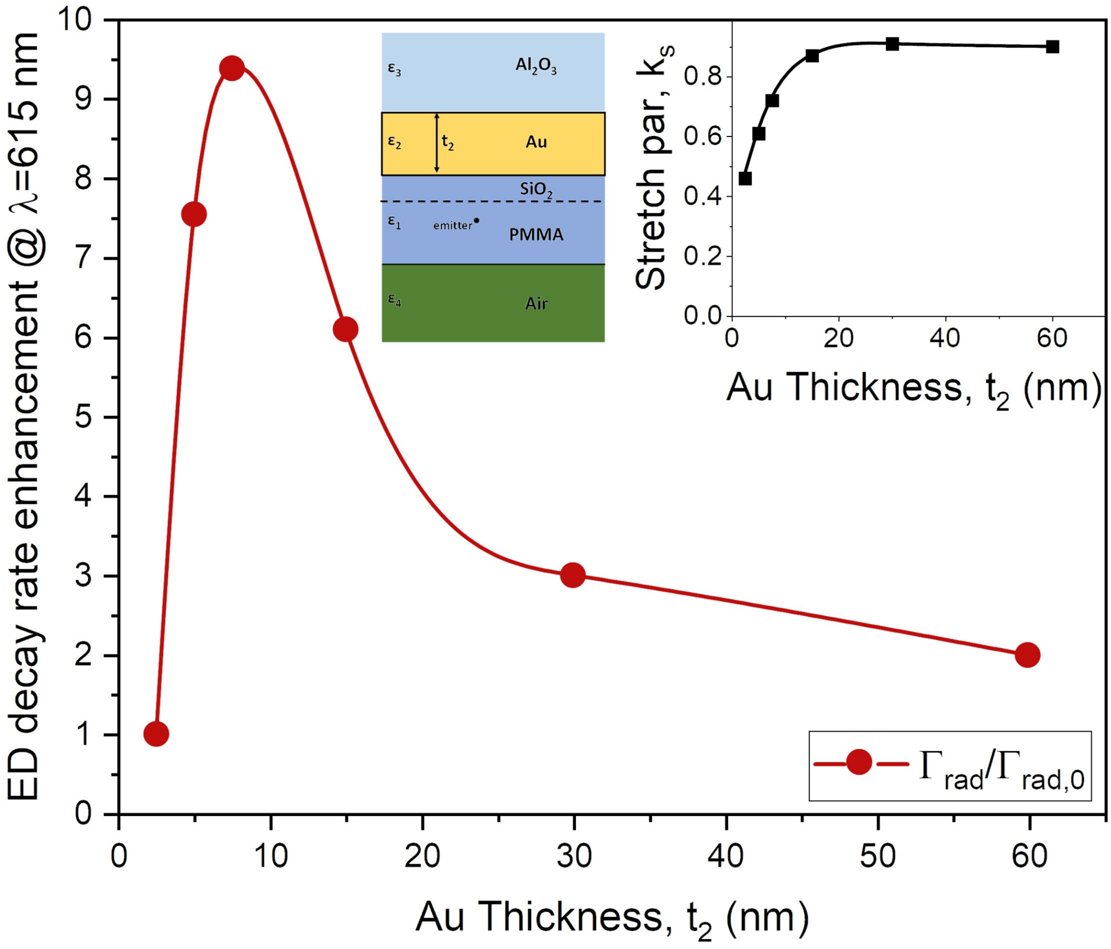

Figure S6. Radiative decay rate enhancement ($\Gamma_{rad}/\Gamma_{rad,0}$, with $\Gamma_{rad,0}$ being the radiative decay rate in a homogeneous PMMA film) for ED emitters at λ = 615 nm computed with the CDO model using the modified Fresnel coefficients as a function of the Au layer thickness (t_2). Since the CDO model accounts for a single distance of the emitter from the interfaces, but the experimental samples have a homogeneous Eu^{3+} distribution in the active layer thickness, the approach described in [Kalinic et al., J. Phys. Chem. C, 119, 6728 (2015)] is followed to account for the emitter box-like distribution along with the sample thickness. The computed decay curves are fitted with a stretched exponential function. In the inset: the stretching parameter of the fitted curves.
